# Endothelial Progenitor Cell Fraction Contained in Bone Marrow-Derived Mesenchymal Stem Cell Populations Impairs Osteogenic Differentiation

**DOI:** 10.1155/2015/659542

**Published:** 2015-09-27

**Authors:** Fabian Duttenhoefer, Rafael Lara de Freitas, Markus Loibl, Gido Bittermann, R. Geoff Richards, Mauro Alini, Sophie Verrier

**Affiliations:** ^1^AO Research Institute Davos, Davos Platz, Switzerland; ^2^Department of Oral and Maxillofacial Surgery, Albert Ludwigs University, Freiburg, Germany; ^3^Medical School of Ribeirão Preto, University of São Paulo, São Paulo, Brazil; ^4^Department of Trauma Surgery, University Medical Center Regensburg, Germany

## Abstract

In bone tissue engineering (TE) endothelial cell-osteoblast cocultures are known to induce synergies of cell differentiation and activity. Bone marrow mononucleated cells (BMCs) are a rich source of mesenchymal stem cells (MSCs) able to develop an osteogenic phenotype. Endothelial progenitor cells (EPCs) are also present within BMC. In this study we investigate the effect of EPCs present in the BMC population on MSCs osteogenic differentiation. Human BMCs were isolated and separated into two populations. The MSC population was selected through plastic adhesion capacity. EPCs (CD34^+^ and CD133^+^) were removed from the BMC population and the resulting population was named depleted MSCs. Both populations were cultured over 28 days in osteogenic medium (Dex^+^) or medium containing platelet lysate (PL). MSC population grew faster than depleted MSCs in both media, and PL containing medium accelerated the proliferation for both populations. Cell differentiation was much higher in Dex^+^ medium in both cases. Real-time RT-PCR revealed upregulation of osteogenic marker genes in depleted MSCs. Higher values of ALP activity and matrix mineralization analyses confirmed these results. Our study advocates that absence of EPCs in the MSC population enables higher osteogenic gene expression and matrix mineralization and therefore may lead to advanced bone neoformation necessary for TE constructs.

## 1. Introduction

Bone is a complex and highly vascularized tissue involving several cell types. Bone development, maintenance, and repair have been shown to be closely dependent on the presence of blood vessels that promote natural bone healing [1, 2]. However, in certain medical conditions, leading to large bone defects (e.g., tumor excision and high impact fractures), the natural repair capacity fails. In particular, in the field of oral and maxillofacial surgery, where comparatively small but anatomically complex bones are affected, reconstruction in terms of an esthetic and functional outcome is often difficult to achieve [3]. To treat defects such as osteoporosis and bisphosphonate-related osteonecrosis of the jaw (BRONJ), cancellous and cortical autologous bone grafts are the gold standard [4]. However, apart from limited bone availability and many times second surgery site complications such as donor side morbidity, possible fracturing of the donor bone may occur [5].

To overcome the various drawbacks of the autologous bone grafts, alternative treatments have been envisaged. Notably, tissue engineering approaches are aiming to reconstruct the missing tissue using cell-based strategies in association with a biomaterial. Bone marrow is a natural and easily available source of stem cells. Bone marrow aspirates are considered to be the most favorable source of mesenchymal stem cells (MSCs) to promote new bone formation [6]. In previous clinical studies, conducted by the authors, successful long-term survival rates of dental implants in MSC-based regenerated bone were shown [7]. In a randomized split-mouth study, MSCs in combination with a bone substitute material showed high implant survival rates similar to those obtained with autologous bone grafts [8].

Large vertical or even critical sized bone defects remain a clinical challenge and the hypothesis that MSCs alone may respond to the local microenvironment of bony defects and thereby promote craniofacial defect regeneration is still at the centre of debate [9].

To date, no specific MSC markers have been identified [10, 11]. Typically, MSCs are enriched from the bone marrow mononucleated cells (BMC)* via* selection of the plastic adherent fibroblastoid cell fraction [12]. Under appropriate experimental conditions, MSCs show a high proliferation rate* in vitro* [6] and can differentiate into bone, cartilage, adipose tissue, and hematopoietic-supportive stromal cells [13]. Recruitment, proliferation, and differentiation of MSCs into mature osteoblasts are regulated by many factors including cytokines, systemic hormones, growth factors, and other regulators [14]. These factors are released to some extent by the osteoblastic cells themselves but also by cells that are part of the tightly connected vascular system, such as endothelial cells [15, 16] or pericytes [17, 18]. It is widely accepted that there is communication between endothelial cells and osteoblastic cells in order to coordinate the formation of blood vessel as well as the differentiation of bone forming cells to regulate bone turnover. Several studies report interactions between osteoblasts or MSCs and endothelial cells. They demonstrated the formation of microvessel-like structures and cell to cell communication through gap junctions [19, 20]. On the other hand, a notable variety of results have been gathered on the influence of endothelial cells on osteoblastic differentiation [21, 22]. This conflicting evidence might be due to the disparity of cell types, cell origin, and experimental set-ups. Most of these studies are using MSCs isolated from BMC fraction by their adherence ability to cell culture plastic. However, amongst the heterogeneous population of BMC, some hematopoietic stem cells also bear the ability to adhere to plastic [23]. In particular CD34 and CD133 positive cell fractions (CD34^+^, CD133^+^) have been identified and are known to give rise to endothelial cells* in vitro* [19, 24]. Postnatal regeneration and neoformation of vessels result from migration and differentiation of lineage committed progenitor cells [25, 26]. This process has been identified as the key mechanism to heal injury in most tissues [27] as, for example, in bone healing [17].

The aim of our study was to investigate the influence of CD34^+^ and CD133^+^ EPCs, contained in the full heterogeneous BMC population, on the osteogenic potential of MSCs. For this purpose, the osteogenic potential of the complete BMC population (called MSC postamplification) was compared with bone marrow samples that have been depleted from all CD34^+^ and CD133^+^ cells (called depleted MSCs). Osteogenic differentiation was induced using either a classical osteogenic medium (containing dexamethasone) or medium containing autologous growth factors (PL) that was shown to promote MSCs differentiation [19, 28].

## 2. Materials and Methods

### 2.1. Cell Culture Media

Iscove's Modified Dulbecco's Medium (IMDM), Fetal Calf Serum (FSC), Nonessential Amino Acids (NEAA), and antibiotics (PenStrep, PS) were purchased from Gibco/Invitrogen Life Technologies (Zug, Switzerland). Basic Fibroblast Growth Factor (bFGF) was purchased from R&D Biosystems (Minneapolis, MN, USA), and ascorbic acid, *β*-glycerophosphate, and dexamethasone were purchased from Sigma-Aldrich (Hamburg, Germany):

 Basic medium: IMDM, 100 U/mL PenStrep (IMDM-PS), 10% FCS, 1% NEAA, and bFGF (5 ng/mL).
 Osteogenic medium: IMDM-PS, 10% FCS, 0.1 mM ascorbic acid, 10 nM dexamethasone, and 10 mM *β*-glycerophosphate.
 PL medium: IMDM-PS, 5% FCS, and 5% PL growth factors.

### 2.2. Bone Marrow

Human bone marrow (BM) samples (20 mL) were obtained from patients undergoing routine orthopaedic surgery upon informed consent and according to Inselspital Bern (Switzerland) ethical commission's guideline (KEK Bern 126/03).

Bone marrow aspirates were obtained from 5 donors (44 to 83 years old, with an average age of 62 years: 4 males and 1 female) in CPDA-containing Sarstedt S-Monovettes (Sarstedt, Nümbrecht, Germany) and processed within 24 hours after harvesting [29].

### 2.3. Cell Populations

*Bone Marrow Mononucleated Cells (BMCs)*. BMCs were isolated from bone marrow aspirates as previously reported [30]. After homogenization, BM aspirates were diluted 1 : 4 with IMDM containing 5% (v/v) FCS and mononucleated cells were separated on a Histopaque-1077 (Sigma-Aldrich) density gradient. Samples were centrifuged at 800 g for 20 minutes. The low-density mononucleated cell interphase was collected and washed twice in 5 mL of IMDM containing 10% FCS, followed by centrifugation at 400 g for 15 minutes. Subsequently, BMCs were further processed for cell selection.

*Mesenchymal Stem Cells (MSCs)*. MSCs were further isolated from total BMC through their plastic adhesion capacity. 16 × 10^6^ cells were seeded in 300 cm^2^ cell culture flask in presence of basic medium and let to adhere for 4 days as described before [29]. After 4 days, nonadherent cells were removed and fresh medium was added. After the first cell amplification step (first passage), cells were called MSCs.

*Endothelial Progenitor Cells (EPCs)*. EPCs were selected from the BMCs population by MiniMAC Magnetic Microbead System (Miltenyi Biotec) using CD34 and CD133 specific antibodies according to manufacturer's instructions. Selected CD34 and CD133 positive cells (CD34^+^ and CD133^+^) were frozen for further experiments.

*EPC-Depleted BMCs (Depleted MSCs) [31]*. After removal of CD34^+^ cells and CD133^+^ cells from the BMCs samples (see “Endothelial Progenitor Cells (EPCs)” section), the resulting cell population was named depleted MSCs.

### 2.4. Platelet Lysate Preparation

Platelet Lysate growth factors (PL) and Platelet Rich Plasma (PRP) were prepared from platelet concentrates, as described earlier [19, 32]. Platelet bags were obtained from the blood bank of Kantonsspital Graubünden in Chur in accordance with the current ethical laws of Switzerland. The platelet bags contain a standardized platelet density (5 times higher than normal), obtained through blood apheresis. We further increased the platelet density by a centrifugation at 2000 g for 7 minutes, followed by resuspension of the pellet in half of the original platelet-bag volume. Phosphate buffered saline (PBS) was used for the PL preparations, while original plasma was used for the PRP preparations to obtain a final concentration 10 times higher than normal blood (2.5 million (±10%) platelets/*μ*L). As expected, the concentrations we obtained were about 10 times higher than the range measured in blood plasma (data not shown). In order to avoid two levels of interindividual variations (bone marrow donors* versus* platelet donors), PL and PRP samples were pooled from three different platelet concentrates and randomly matched [33, 34].

### 2.5. Cell Expansion

Cells (MSCs and depleted MSCs) were seeded at the density of 0.9 × 10^6^ mononucleated cells per 300 cm^2^ T-flask (Techno Plastic Products AG, Trasadingen, Switzerland) in basic media [35]. Medium was changed every 3 days, and cells were subcultured 1 : 4. Cells in passages 2–4 were subsequently used. MSCs and depleted MSCs were further cultured in the exact same conditions.

### 2.6. Cell Differentiation

MSCs or depleted MSCs were seeded at the density of 10′000 cells/cm^2^ in 24-well plates in triplicate for each donor and each analysis. Cells were cultured for 28 days in osteogenic medium and PL medium as described above [36].

### 2.7. Cell Growth (DNA Quantification Assay)

Cell growth was determined as described by Labarca and Paigen [37] after 1, 7, 14, 21, and 28 days of culture in either osteogenic medium or PL medium. Briefly, DNA was quantified by measuring the binding of Hoechst 33258 (Polysciences Inc., 09460) to the DNA helix after cells overnight digestion at 56°C in a proteinase K solution (0.5 mg/mL in 3.36 mg/mL disodium-EDTA-PBS). After appropriate dilution of the samples in Dulbecco's phosphate buffered saline (DPBS) containing 0.1% (v/v) H33258, the bound fluorescence was measured using a PE HTS 7000 Bio Assay Reader at 360 nm excitation and 465 nm emission wavelength.

### 2.8. Gene Expression Analysis

*RNA Isolation and Reverse Transcription.* Total RNA was extracted from cells monolayers at different time points (days 1, 7, 14, 21, and 28) using TRI-Reagent (MRC Inc., TR-118) according to the manufacturer's instructions (Molecular Research Center, Cincinnati, Ohio). cDNA was synthetized from 1 *μ*g of total RNA using TaqMan reverse transcription reagents (Applied Biosystems, Foster City, CA) with random hexamer primers.

*Real-Time Polymerase Chain Reaction (PCR).* Real-Time Polymerase Chain Reaction (PCR) was performed on a 7500 Real-Time PCR System (Applied Biosystems). Genes of interest were detected using specific oligonucleotide primers and TaqMan probes (Microsynth, Balgach, Switzerland) or Assays-on-Demand (Applied Biosystems) as specified in Table 1. Eukaryotic 18S (Applied Biosystems) was used as a housekeeping gene. PCR conditions were 95°C for 10 min, followed by 42 cycles of amplification at 95°C for 15 sec and 60°C for 1 min using the GeneAmp 5700 Sequence Detection System (Applied Biosystems). Gene expression was analysed according to the ^ΔΔ^CT method, with expression normalized to the corresponding reference (specified on each graph) at the same time point.

### 2.9. ALP Activity Assay

Samples for ALP activity measurement were harvested on days 1, 7, 14, 21, and 28. After medium removal and a washing step with PBS, 1 mL of 0.1% Triton-X in 10 mM Tris-HCl (pH 7.4) was added to the cell monolayers and incubated for 4 h at 4°C on a gyratory shaker [38]. ALP activity was assessed by measuring the p-nitrophenol production during 15 min incubation at 37°C with p-nitrophenyl phosphate as substrate (Sigma Kit number 104) on a Perkin Elmer Bio Assay Reader HTS 7000.

### 2.10. ^45^Ca^2+^ Incorporation Assay

Matrix mineralization was estimated by the incorporation of ^45^Ca^2+^ into the extracellular matrix. 1.25 *μ*Ci/mL isotope (Amersham CES3, Amersham, UK) was diluted in pure IMDM, added to each well, and incubated at 37°C o/n [38]. After medium removal and 3 washes in PBS, 0.5 mL of 70% formic acid was added to each well and incubated at 65°C for 1 h. The formic acid solution was transferred to a scintillation tube containing 3.5 mL of scintillation liquid (OptiPhase HiSafe'3, Perkin Elmer, Waltham, MA, USA) and the radioactivity was measured after 28 days of culture using a Wallac 1414 WinSpectral Liquid Scintillation Counter (Perkin Elmer).

### 2.11. Statistical Analyses

Statistical analyses were performed using the software package SPSS. Data were tested for normal distribution using Shapiro-Wilk test. Consequently, data were analyzed using a general linear model with repeated measures. *p* values were corrected by Bonferroni's method. All experiments were done using 5 different donors and in triplicate for each donor. Significant values were defined as ^*∗*^ *p* < 0.05 and ^*∗∗*^ *p* < 0.01.

## 3. Results

### 3.1. Cell Growth

**Figure 1:**
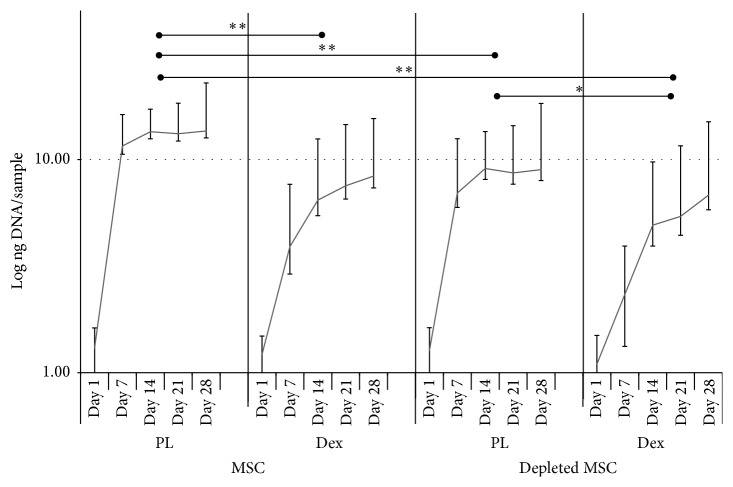
Cell proliferation. MSCs or depleted MSCs were cultured in presence of medium containing Dex or PL. DNA quantification was performed at different time points over a period of 28 days.

Cell growth was assessed by DNA quantification. MSC and depleted MSC populations were seeded into tissue culture plastic wells and cultured in presence of PL- or dexamethasone-containing medium. As shown in Figure 1 (note the logarithmic scale of the *y*-axis), both cell populations showed a typical cell growth profile, starting with an exponential growth phase, reaching a plateau of proliferation. In both cell populations, this plateau was reached around day 14 when cells were cultured in medium containing dexamethasone. In the presence of PL, cells grew significantly faster for both cell populations (*p* < 0.01 for MSC and *p* < 0.05 for depleted MSC) than in dexamethasone-containing medium. In PL medium, the MSCs population showed a significantly higher cell proliferation rate when compared to the depleted MSCs in the same conditions (*p* < 0.01) (Figure 1).

### 3.2. Osteogenic Gene Expression

**Figure 2:**
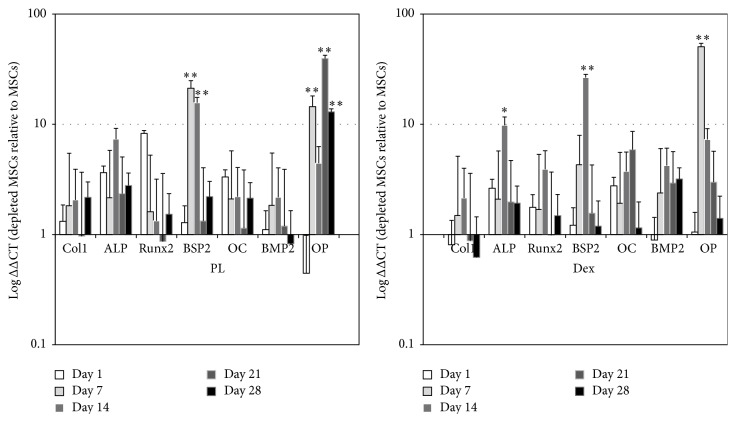
Osteogenic gene expression analysis. Osteoblastic marker genes were analyzed by real-time RT-PCR on the two cell populations. Results are expressed in expression of fold changes in depleted MSC relative to MSC.

In addition to cell proliferation, the osteogenic differentiation of depleted MSCs and MSCs was looked at. Cells were cultured in either PL-containing medium or dexamethasone-containing medium during the course of 28 days. RNA samples were extracted at different time points of culture, and real-time RT-PCR was performed for osteogenic marker genes. Results are presented as relative gene expression in depleted MSCs relative to MSCs (^ΔΔ^CT). When depleted MSCs were cultured in PL medium, all studied osteoblastic genes were upregulated in comparison to the MSC population cultured in the same condition. This finding was consistently observed at each time point (Figure 2). In addition, the same trend of upregulation was observed for all genes when cells were cultured in classical osteogenic medium (Dex^+^). Most of the genes were significantly highly expressed in depleted MSCs compared to MSCs in both media (PL and Dex). Of particular interest was the significant upregulation of Bone Sialoprotein 2 (BSP2) and Osteopontin (OP) in both media (*p* < 0.01) and ALP in presence of dexamethasone (*p* < 0.05). Gene expression is reported as log fold regulation in the depleted MSC population relative to the MSC population at the same time point in Figure 2.

### 3.3. ALP Activity

**Figure 3:**
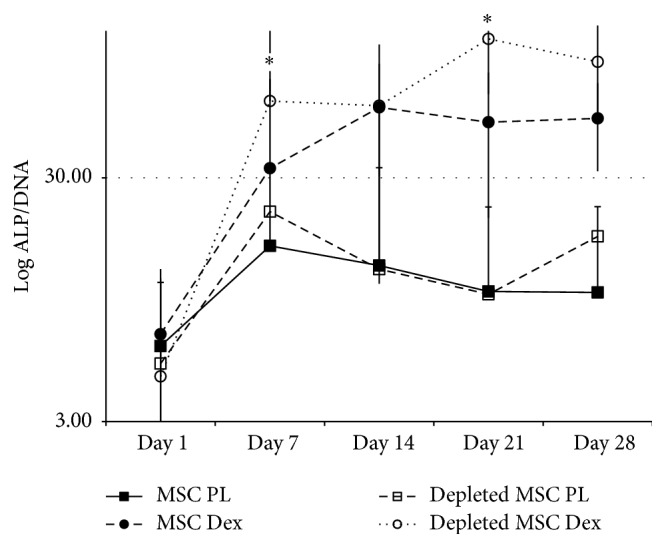
ALP activity of cells cultured in presence of Dex^+^ showed a highly significant level compared to PL medium (*p* < 0.01) in parallel; ALP level of activity was more elevated in depleted MSCs compared to MSCs (*p* < 0.05). ALP activity values were corrected to cell numbers (ALP/DNA).

Looking at the ALP activity (Figure 3), both cell populations (MSCs and depleted MSCs) showed a low peak of alkaline phosphatase activity by day 7 in presence of PL medium, with a significantly higher peak for depleted MSC compared to MSC. On the contrary, a high peak of alkaline phosphatase activity was obtained in Dex^+^ medium for both cell populations. This activity level was significantly higher in depleted MSCs when compared to MSCs (*p* < 0.01 for the overall time points).

### 3.4. ^45^Ca^2+^ Incorporation

**Figure 4:**
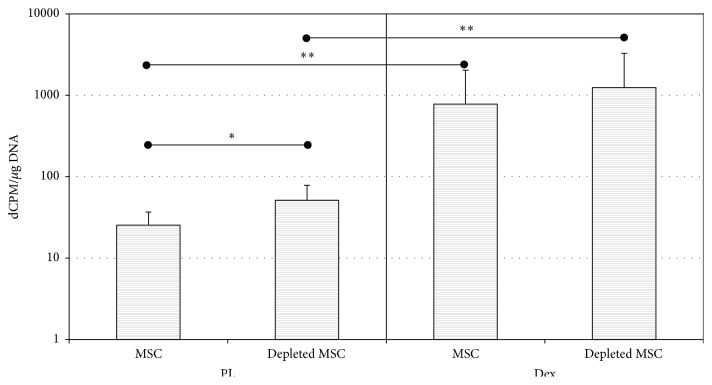
^45^Ca^2+^ was measured after 28 days of culture in PL or Dex^+^ medium. Results are presented in dCPM related to cell number. Matrix mineralization was found higher in presence of Dex^+^ (*p* < 0.01) compared to PL, while depleted MSCs showed better ability to mineralize their matrix compared to MSCs (*p* < 0.05 in PL).

Matrix mineralization was followed by ^45^Ca^2+^ incorporation (Figure 4). Depleted MSCs and MSCs populations were cultured during 28 days in presence of either PL-containing medium or classical osteogenic medium (Dex) (Figure 4). Interestingly, depleted MSCs cultured in PL medium showed a high incorporation of ^45^Ca^2+^in comparison to MSCs after 28 days of cell culture. The presence of dexamethasone in osteogenic medium resulted in a further increase of calcium incorporation in both depleted MSCs and MSCs (both *p* < 0.01). Matrix mineralization obtained by the use of depleted MSCs was higher than that for MSCs in PL medium (*p* < 0.05) and appears to be higher in osteogenic medium (missing statistical significance).

### 3.5. EPC Population Contained in Full MSC

**Figure 5:**
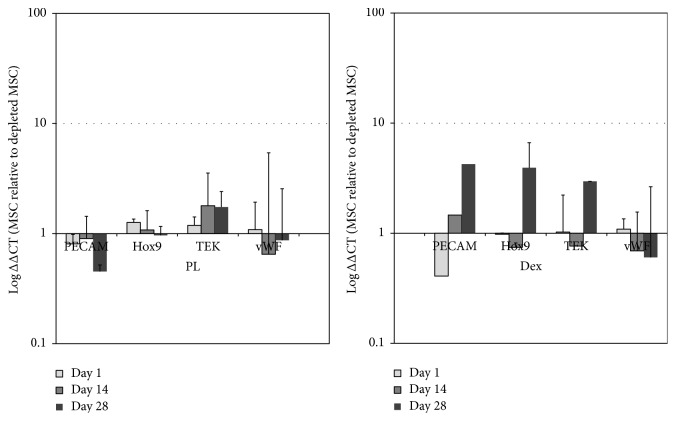
Endothelial marker genes analysis in depleted MSCs and MSCs after 28 days of culture was performed by real-time RT-PCR. No significant differences in gene expression pattern could be observed between MSC and depleted MSC populations. The results are expressed in log fold changes in MSCs relative to depleted MSCs (^ΔΔ^CT).

To follow the prevalence of the EPC fractions contained in the MSC population, we performed gene expression analysis for endothelial specific marker genes over a period of 28 days (Figure 5). The genes expression in MSCs is reported as fold changes of the gene expression relative to the same genes in depleted MSCs. Values are shown in logarithmic scale. As seen in Figure 5, no significant changes in gene expression pattern were obtained for MSC compared to the depleted MSC population, showing that the EPCs present in the MSCs population did not differentiate towards a mature endothelial cell phenotype. The same trend of results was obtained in both culture media (PL and Dex).

## 4. Discussion

In the present study, we investigated the influence of the naturally coresident endothelial progenitor cells in bone marrow on the proliferation and osteogenic differentiation of MSCs. MSCs (selected through their plastic adhesion ability) were compared to EPC-depleted BMC population (depleted MSCs). These two cell populations were cultured in presence of classical osteogenic medium (containing *β*-glycerophosphate, ascorbic acid, and dexamethasone) or in presence of medium (containing *β*-glycerophosphate, ascorbic acid, and PL). During 28 days of cell culture in either of these media, we followed cell proliferation, cell osteogenic differentiation, alkaline phosphatase activity, and matrix mineralization. We found that the osteogenic potential of the EPC-depleted cell population was higher than that for MSC. On the opposite side, the MSC population grew significantly faster than the depleted MSCs population in the presence of PL medium when compared to the Dex^+^ medium.

MSCs were first described by Friedenstein et al. [28] and are characterized by the ability to differentiate* in vitro* into the three mesenchymal lineages, that is, cartilage, fat, and, in our case, bone [13]. The classical osteogenic differentiation of human MSCs [35] requires incubation of cell monolayers with ascorbic acid, *β*-glycerophosphate, and dexamethasone (added to medium containing FCS), resulting in increased alkaline phosphatase activity followed by calcium deposition.

Intense investigations on MSC selection have been performed. The most common method consists in the MSCs selection through their ability to adhere to cell culture plastic, followed by serial passaging to reduce the presence of remaining hematopoietic cells. The use of monoclonal antibodies in order to preselect MSCs with the use of surface marker such as CD90 and CD105 (i.e., positive selection) [39, 40] is also deeply investigated. On the contrary, a negative selection approach can also be used. In this case, other cell types, such as hematopoietic cells, usually removed [41] through different adhesion properties can be sorted from the BMC pool using CD34 and CD133 specific antibodies.

Hematopoietic stem cells gained increasing attention for their potential use in regenerative medicine and tissue engineering, essentially the CD34^+^ and CD133^+^ endothelial progenitor cells [42, 43]. Stem cell niches have been described so far for a number of tissue types such as the hair follicle, intestine, and the bone marrow [44, 45]. The two distinct niches of hematopoietic stem cells (HSCs) in bone marrow are the endosteal niche with HSC in close contact with osteoblasts and the perivascular niche where HSCs are found close to the sinusoids. This conformation might serve as a good example for the complexity of the niche functional concept [45]. In the endosteal niche signaling events between osteoblasts and HSCs play a crucial role in maintenance and activation of stem cells [46]. There is increasing evidence to indicate that MSC populations are heterogeneous with coexisting subsets having varying potency. This applies to bone marrow MSCs as well as those from other tissues [47].

Taking niche signaling processes into account, a variety of studies were conducted during the past decade looking at the influence of endothelial cell coculture with osteoblastic cells and/or MSC. And even if the effects described concerning the osteogenic differentiation of MSC are relatively divergent [20, 48], the positive effect of this coculture on MSCs and osteoblastic cells proliferation is quite consistent [22, 49]. However, in all these studies, endothelial cells (EC) (mainly HUVEC) were added to the MSC population. In our case, no endothelial cells were added to the cultures. Instead, endothelial progenitor cells were left in their natural niche microenvironment containing MSCs and compared with BMCs that were depleted from their natural EPC content (depleted MSCs).

In previous investigations, PL showed positive effects on endothelial progenitor cell proliferation [32] and on gene expression of pericyte markers in MSCs and depleted MSCs [31]. In contrast, in the present work, no significant differences were depicted between MSCs and depleted MSCs endothelial specific gene expression when cultured in either PL or osteogenic medium. Nevertheless, the overall osteogenic gene expression as well as matrix mineralization potential of the depleted MSC population was higher than for MSCs. In the present investigation, EPCs remained in their niche environment along with MSCs and therefore provided them with a specific microenvironment protecting the stemness characteristics of MSCs [50]. In the depleted MSCs population, on the other hand, the remaining MSCs may have lost such protective paracrine and direct cell contact mechanisms and could therefore be more susceptible to osteogenic differentiation. Similar mechanisms were described previously, where the cross talk of bone marrow-derived EPCs (BM-EPC) and MSCs through paracrine and direct cell contact mechanisms could modulate the angiogenic response [51].

In synopsis with the present data, it is tempting to speculate that the degree of differentiation and therefore the maturity of EPCs could play an important role in their influence on MSCs osteogenic differentiation. This is supported by the study of Loibl et al. 2014 that indicates EPC differentiation into mature EC by direct cell-cell contact with MSCs [31]. Furthermore, there is evidence that the presence of PL induced cell growth of EPC in EPC-MSC coculture supports a pericyte-like differentiation of MSCs in both MSC and EPC-depleted MSC populations [29, 31]. Moreover, it was shown that the addition of MSC promotes stable neovascularization in EPC-derived tube formation* in vivo* [52]. Still, the ideal ratio of EPC for early neovascularization is controversially debated in the literature. Previous* in vitro* experiments from our group found the ratio of 50/50 of MSC and EPC ideal [29]. These results were corroborated* in vivo*, where the highest number of vessels in the center of scaffolds, implanted subcutaneously in nude mice, was found in 50% MSC + 50% EPC proportion [53]. On the contrary, Fu et al., 2015, concluded that the ratio of 75% EPC + 25% MSC in modified calcium polyphosphate constructs showed the highest expression of osteogenic and angiogenic markers, whereas the degree of EPC maturation still remains unclear [54].

A limitation of this work certainly could be the average age of the bone marrow donors (62 years). Siegel et al. [55] showed the influence of gender and/or age (and age-related medication intake) on several MSCs characteristics such as time of doubling population, while no correlation was found between donor's age or gender and the expression level of some stemness related genes (e.g., Oct4 or Nanog). In another study, using MSCs from late adult patients' bone marrow (52 to 92 years old), Leskelä et al. [56] suggested that the osteogenic differentiation potential does not decrease with age. Likewise, Dexheimer et al. reported the influence of the cell proliferation status rather than the age or gender of the patient on the multilineage differentiation potential of MSCs [57]. In a study published by Herrmann et al. [58], no correlation was observed between age or gender and the percentage of CD133/CD34 double positive EPCs present in bone marrow samples from patients with an average age of 63 years. In this present work, we compared the differentiation potential of MSCs with their corresponding depleted MSCs (same donor) and could demonstrate the effect of EPCs on the MSCs growth and differentiation.

In detail, our results indicate that absence of EPCs in MSC population enables higher osteogenic gene expression and matrix mineralization and therefore may lead to earlier new bone formation. Nevertheless, the application of cells in bone tissue engineered constructs demands the support of a functional blood supply. Therefore our results may lead to novel approaches in cell seeding to develop vascularized bone tissue engineered scaffolds, such as selective, prioritized, or time dependent seeding of different cell types. Still, further investigations on the mechanisms by which CD34^+^/CD133^+^ EPC influence MSC osteogenic differentiation as well as the influence of EPC maturation in this process are necessary.

## Figures and Tables

**Table 1:** Genes of interest were detected by Polymerase Chain Reaction (PCR) using specific oligonucleotide primers, TaqMan probes, or Assays-on-Demand. Eukaryotic 18S was used as a housekeeping gene.

Microsynth (target gene sequence (5′→3′))	Applied Biosystems
Bone marker genes	Bone marker genes ALP: Hs00758162_m1 Endothelial marker genes PECAM-I: HS01065282_m1 vWF: Hs00169795_m1 TEK: Hs00176096_m1 Flt1: Hs00176573_m1 KDR: Hs00176676_m1 HOX9: Hs00365956_m1 Housekeeping gene 18s: 4319413E
Collagen I*α*1 Forw CCC TGG AAA GAA TGG AGA TGA T Rev ACT GAA ACC TCT GTG TCC CTT CA Probe CGG GCA ATC CTC GAG CAC CCT	
Osteonectin	
Forw ATC TTC CCT GTA CAC TGG CAG TTC Rev CTC GGT GTG GGA GAG GTA CC Probe CAG CTG GAC CAG CAC CCC ATT GAC	
BMP-2	
Forw AAC ACT GTG CGC AGC TTC C Rev CTC CGG GTT GTT TTC CCA C Probe CCA TGA AGA ATC TTT GGA AGA ACT ACC AGA AAC TG	
Osteopontin	
Forw CTC AGG CCA GTT GCA GCC Rev CAA AAG CAA ATC ACT GCA ATT CTC Probe AAA CGC CGA CCA AGG AAA ACT CAC TAC C	
Runx2	
Forw AGC AAG GTT CAA CGA TCT GAG AT Rev TTT GTG AAG ACG GTT ATG GTC AA Probe TGA AAC TCT TGC CTC GTC CAC TCC G	
BSP II	
Forw TGC CTT GAG CCT GCT TCC Rev GCA AAA TTA AAG CAG TCT TCA TTT TG Probe CTC CAG GAC TGC CAG AGG AAG CAA TCA	

## References

[1] Fuchs S., Hofmann A., Kirkpatrick C. J. (2007). Microvessel-like structures from outgrowth endothelial cells from human peripheral blood in 2-dimensional and 3-dimensional co-cultures with osteoblastic lineage cells. *Tissue Engineering*.

[2] Kanczler J. M., Oreffo R. O. C. (2008). Osteogenesis and angiogenesis: the potential for engineering bone. *European Cells and Materials*.

[3] Schmitz J. P., Hollinger J. O. (1986). The critical size defect as an experimental model for craniomandibulofacial nonunions. *Clinical Orthopaedics and Related Research*.

[4] Tong D. C., Rioux K., Drangsholt M., Beirne O. R. (1998). A review of survival rates for implants placed in grafted maxillary sinuses using meta-analysis. *International Journal of Oral and Maxillofacial Implants*.

[5] Beirne J. C., Barry H. J., Brady F. A., Morris V. B. (1996). Donor site morbidity of the anterior iliac crest following cancellous bone harvest. *International Journal of Oral and Maxillofacial Surgery*.

[6] Niemeyer P., Fechner K., Milz S. (2010). Comparison of mesenchymal stem cells from bone marrow and adipose tissue for bone regeneration in a critical size defect of the sheep tibia and the influence of platelet-rich plasma. *Biomaterials*.

[7] Duttenhoefer F., Hieber S. F., Stricker A., Schmelzeisen R., Gutwald R., Sauerbier S. (2014). Follow-up of implant survival comparing ficoll and bone marrow aspirate concentrate methods for hard tissue regeneration with mesenchymal stem cells in humans. *BioResearch Open Access*.

[8] Rickert D., Vissink A., Slot W. J., Sauerbier S., Meijer H. J. A., Raghoebar G. M. (2014). Maxillary sinus floor elevation surgery with BioOss mixed with a bone marrow concentrate or autogenous bone: test of principle on implant survival and clinical performance. *International Journal of Oral and Maxillofacial Surgery*.

[9] Yang Y., Hallgrimsson B., Putnins E. E. (2011). Craniofacial defect regeneration using engineered bone marrow mesenchymal stromal cells. *Journal of Biomedical Materials Research*.

[10] Caplan A. I., Correa D. (2011). The MSC: an injury drugstore. *Cell Stem Cell*.

[11] Bianco P., Robey P. G., Simmons P. J. (2008). Mesenchymal stem cells: revisiting history, concepts, and assays. *Cell Stem Cell*.

[12] Pittenger M. F. (2008). Mesenchymal stem cells from adult bone marrow. *Methods in Molecular Biology*.

[13] Pittenger M. F., Mackay A. M., Beck S. C. (1999). Multilineage potential of adult human mesenchymal stem cells. *Science*.

[14] Lian J. B., Stein G. S. (1995). Development of the osteoblast phenotype: molecular mechanisms mediating osteoblast growth and differentiation. *The Iowa Orthopaedic Journal*.

[15] Tepper O. M., Capla J. M., Galiano R. D. (2005). Adult vasculogenesis occurs through in situ recruitment, proliferation, and tubulization of circulating bone marrow-derived cells. *Blood*.

[16] Street J., Bao M., DeGuzman L. (2002). Vascular endothelial growth factor stimulates bone repair by promoting angiogenesis and bone turnover. *Proceedings of the National Academy of Sciences of the United States of America*.

[17] Matsumoto T., Kawamoto A., Kuroda R. (2006). Therapeutic potential of vasculogenesis and osteogenesis promoted by peripheral blood CD34-positive cells for functional bone healing. *The American Journal of Pathology*.

[18] Jones A. R., Clark C. C., Brighton C. T. (1995). Microvessel endothelial cells and pericytes increase proliferation and repress osteoblast phenotypic markers in rat calvarial bone cell cultures. *Journal of Orthopaedic Research*.

[19] Verrier S., Meury T. R., Kupcsik L., Heini P., Stoll T., Alini M. (2010). Platelet-released supernatant induces osteoblastic differentiation of human mesenchymal stem cells: potential role of BMP-2. *European Cells and Materials*.

[20] Villars F., Guillotin B., Amédée T. (2002). Effect of HUVEC on human osteoprogenitor cell differentiation needs heterotypic gap junction communication. *The American Journal of Physiology—Cell Physiology*.

[21] Bianchi G., Banfi A., Mastrogiacomo M. (2003). Ex vivo enrichment of mesenchymal cell progenitors by fibroblast growth factor 2. *Experimental Cell Research*.

[22] Xue Y., Xing Z., Hellem S., Arvidson K., Mustafa K. (2009). Endothelial cells influence the osteogenic potential of bone marrow stromal cells. *BioMedical Engineering OnLine*.

[23] Gordon M. Y., Levičar N., Pai M. (2006). Characterization and clinical application of human CD34^+^ stem/progenitor cell populations mobilized into the blood by granulocyte colony-stimulating factor. *Stem Cells*.

[24] Asahara T., Masuda H., Takahashi T. (1999). Bone marrow origin of endothelial progenitor cells responsible for postnatal vasculogenesis in physiological and pathological neovascularization. *Circulation Research*.

[25] Zammaretti P., Zisch A. H. (2005). Adult ‘endothelial progenitor cells’: renewing vasculature. *International Journal of Biochemistry and Cell Biology*.

[26] Khurana R., Simons M. (2003). Endothelial progenitor cells: precursors for angiogenesis. *Seminars in Thoracic and Cardiovascular Surgery*.

[27] Tepper O. M., Sealove B. A., Murayama T., Asahara T. (2003). Newly emerging concepts in blood vessel growth: recent discovery of endothelial progenitor cells and their function in tissue regeneration. *Journal of Investigative Medicine*.

[28] Friedenstein A. J., Chailakhyan R. K., Latsinik N. V., Panasyuk A. F., Keiliss-Borok I. V. (1974). Stromal cells responsible for transferring the microenvironment of the hemopoietic tissues. Cloning in vitro and retransplantation in vivo. *Transplantation*.

[29] Duttenhoefer F., de Freitas R. L., Meury T. (2013). 3D scaffolds co-seeded with human endothelial progenitor and mesenchymal stem cells: evidence of prevascularisation within 7 days. *European Cells & Materials*.

[30] Bruder S. P., Jaiswal N., Haynesworth S. E. (1997). Growth kinetics, self-renewal, and the osteogenic potential of purified human mesenchymal stem cells during extensive subcultivation and following cryopreservation. *Journal of Cellular Biochemistry*.

[31] Loibl M., Binder A., Herrmann M. (2014). Direct cell-cell contact between mesenchymal stem cells and endothelial progenitor cells induces a pericyte-like phenotype *in vitro*. *BioMed Research International*.

[32] Lippross S., Loibl M., Hoppe S. (2011). Platelet released growth factors boost expansion of bone marrow derived CD34^+^ and CD133^+^ endothelial progenitor cells for autologous grafting. *Platelets*.

[33] Herrmann M., Binder A., Menzel U., Zeiter S., Alini M., Verrier S. (2014). CD34/CD133 enriched bone marrow progenitor cells promote neovascularization of tissue engineered constructs *in vivo*. *Stem Cell Research*.

[34] Lubkowska A., Dolegowska B., Banfi G. (2012). Growth factor content in PRP and their applicability in medicine. *Journal of Biological Regulators and Homeostatic Agents*.

[35] Jaiswal N., Haynesworth S. E., Caplan A. I., Bruder S. P. (1997). Osteogenic differentiation of purified, culture-expanded human mesenchymal stem cells in vitro. *Journal of Cellular Biochemistry*.

[36] Lucarelli E., Beccheroni A., Donati D. (2003). Platelet-derived growth factors enhance proliferation of human stromal stem cells. *Biomaterials*.

[37] Labarca C., Paigen K. (1980). A simple, rapid, and sensitive DNA assay procedure. *Analytical Biochemistry*.

[38] Alini M., Carey D., Hirata S., Grynpas M. D., Pidoux I., Poole A. R. (1994). Cellular and matrix changes before and at the time of calcification in the growth plate studied in vitro: arrest of type X collagen synthesis and net loss of collagen when calcification is initiated. *Journal of Bone and Mineral Research*.

[39] Campioni D., Lanza F., Moretti S. (2003). Functional and immunophenotypic characteristics of isolated CD105^+^ and fibroblast^+^ stromal cells from AML: implications for their plasticity along endothelial lineage. *Cytotherapy*.

[40] Flores-Figueroa E., Arana-Trejo R. M., Gutiérrez-Espíndola G., Pérez-Cabrera A., Mayani H. (2005). Mesenchymal stem cells in myelodysplastic syndromes: phenotypic and cytogenetic characterization. *Leukemia Research*.

[41] Baddoo M., Hill K., Wilkinson R. (2003). Characterization of mesenchymal stem cells isolated from murine bone marrow by negative selection. *Journal of Cellular Biochemistry*.

[42] Gordon M. Y. (2008). Stem cells for regenerative medicine—biological attributes and clinical application. *Experimental Hematology*.

[43] Kawamoto A., Asahara T., Losordo D. W. (2002). Transplantation of endothelial progenitor cells for therapeutic neovascularization. *Cardiovascular Radiation Medicine*.

[44] Fuchs E., Tumbar T., Guasch G. (2004). Socializing with the neighbors: stem cells and their niche. *Cell*.

[45] Mitsiadis T. A., Barrandon O., Rochat A., Barrandon Y., De Bari C. (2007). Stem cell niches in mammals. *Experimental Cell Research*.

[46] Kiel M. J., Morrison S. J. (2008). Uncertainty in the niches that maintain haematopoietic stem cells. *Nature Reviews Immunology*.

[47] Lee C. C. I., Christensen J. E., Yoder M. C., Tarantal A. F. (2010). Clonal analysis and hierarchy of human bone marrow mesenchymal stem and progenitor cells. *Experimental Hematology*.

[48] Meury T., Verrier S., Alini M. (2006). Human endothelial cells inhibit BMSC differentiation into mature osteoblasts in vitro by interfering with osterix expression. *Journal of Cellular Biochemistry*.

[49] Bidarra S. J., Barrias C. C., Barbosa M. A., Soares R., Amédée J., Granja P. L. (2011). Phenotypic and proliferative modulation of human mesenchymal stem cells via crosstalk with endothelial cells. *Stem Cell Research*.

[50] Scadden D. T. (2006). The stem-cell niche as an entity of action. *Nature*.

[51] Aguirre A., Planell J. A., Engel E. (2010). Dynamics of bone marrow-derived endothelial progenitor cell/mesenchymal stem cell interaction in co-culture and its implications in angiogenesis. *Biochemical and Biophysical Research Communications*.

[52] Fedorovich N. E., Haverslag R. T., Dhert W. J. A., Alblas J. (2010). The role of endothelial progenitor cells in prevascularized bone tissue engineering: development of heterogeneous constructs. *Tissue Engineering, Part A*.

[53] Hermann J. S., Buser D., Schenk R. K., Cochran D. L. (2000). Crestal bone changes around titanium implants. A histometric evaluation of unloaded non-submerged and submerged implants in the canine mandible. *Journal of Periodontology*.

[54] Fu W.-L., Xiang Z., Huang F.-G. (2015). Coculture of peripheral blood-derived mesenchymal stem cells and endothelial progenitor cells on strontium-doped calcium polyphosphate scaffolds to generate vascularized engineered bone. *Tissue Engineering Part A*.

[55] Siegel G., Kluba T., Hermanutz-Klein U., Bieback K., Northoff H., Schäfer R. (2013). Phenotype, donor age and gender affect function of human bone marrow-derived mesenchymal stromal cells. *BMC Medicine*.

[56] Leskelä H. V., Risteli J., Niskanen S., Koivunen J., Ivaska K. K., Lehenkari P. (2003). Osteoblast recruitment from stem cells does not decrease by age at late adulthood. *Biochemical and Biophysical Research Communications*.

[57] Dexheimer V., Mueller S., Braatz F., Richter W. (2011). Reduced reactivation from dormancy but maintained lineage choice of human mesenchymal stem cells with donor age. *PLoS ONE*.

[58] Herrmann M., Binder A., Menzel U., Zeiter S., Alini M., Verrier S. (2014). CD34/CD133 enriched bone marrow progenitor cells promote neovascularization of tissue engineered constructs in vivo. *Stem Cell Research*.

